# Clinical Stage-Specific Alteration of hsa-miR-424-5p in Milk Small Extracellular Vesicles from Bovine Leukemia Virus Infection to Progression of Enzootic Bovine Leukosis

**DOI:** 10.3390/ani16192976

**Published:** 2026-09-22

**Authors:** Md. Matiur Rahman, Shota Moriyasu, Yuji O. Kamatari, Ayaka Okada, Yasuo Inoshima

**Affiliations:** 1 Laboratory of Food and Environmental Hygiene, Joint Department of Veterinary Medicine, Gifu University, Gifu 501-1193, Japan; matiur.vetmed@gmail.com (M.M.R.); okada.ayaka.i2@f.gifu-u.ac.jp (A.O.); 2 Department of Medicine, Faculty of Veterinary, Animal and Biomedical Sciences, Sylhet Agricultural University, Sylhet 3100, Bangladesh; 3 Institute for Glyco-Core Research (iGCORE), Gifu University, Gifu 501-1193, Japan; kamatari.yuji.f2@f.gifu-u.ac.jp; 4 The United Graduate School of Drug Discovery and Medical Information Sciences, Gifu University, Gifu 501-1193, Japan; 5 Division of Instrumental Analysis, Life Science Research Center, Gifu University, Gifu 501-1193, Japan; 6 Education and Research Center for Food Animal Health, Gifu University (GeFAH), Gifu 501-1193, Japan; 7 Joint Graduate School of Veterinary Sciences, Gifu University, Gifu 501-1193, Japan

**Keywords:** bovine leukemia virus, clinical stage, enzootic bovine leukosis, miRNA, prognosis, milk small EV

## Abstract

Bovine leukemia virus (BLV) is an oncogenic retrovirus responsible for enzootic bovine leukosis (EBL) in cattle. Previous studies indicated that increased levels of the miRNA hsa-miR-424-5p in milk small extracellular vesicles (sEVs) may serve as an EBL biomarker in cattle; however, its alteration across the clinical stages of BLV infection to the onset of EBL remains unclear. This study evaluated the prognostic potential of hsa-miR-424-5p in milk sEVs across distinct clinical stages of BLV infection to EBL onset. qPCR analysis showed that the abundance of hsa-miR-424-5p was lower in milk sEVs from BLV-infected cattle with low and high proviral load (LPVL and HPVL) compared to BLV-uninfected healthy cattle (Control), but was not significant (*p* > 0.05). In contrast, the abundance of hsa-miR-424-5p in milk sEVs was significantly higher in EBL cattle than in the Control and HPVL cattle (*p* < 0.05). Spearman’s correlation analysis revealed a significant inverse association between hsa-miR-424-5p and the percentage of lactate dehydrogenase 2 + 3 (r = −0.28, *p* < 0.001), whereas no significant correlations were observed with other parameters. hsa-miR-424-5p showed the highest discriminative performance in distinguishing BLV-infected cattle from EBL cattle (area under the curve [AUC] = 0.71), followed by Control vs. BLV (AUC = 0.63), indicating statistically meaningful biomarker utility across disease stages. These findings suggest that the amount of hsa-miR-424-5p in milk sEVs increases during the progression from the late stage of BLV infection to EBL onset, demonstrating that hsa-miR-424-5p may serve as a part of the biomarker panel for monitoring BLV progression and predicting EBL onset.

## Simple Summary

Bovine leukemia virus (BLV) is a major infectious disease of cattle that can lead to enzootic bovine leukosis (EBL). The early identification of cattle at risk of developing EBL remains challenging. Small extracellular vesicles (sEVs) in bovine milk contain microRNAs (miRNAs) that may reflect disease progression. In this study, we investigated the abundance of hsa-miR-424-5p in milk sEVs from BLV-uninfected healthy cattle (Control), BLV-infected cattle with low and high proviral load (HPVL), and cattle with EBL. Our results showed that the level of hsa-miR-424-5p was slightly lower in BLV-infected cattle than in Control cattle. However, it was significantly increased in cattle with EBL compared with Control cattle and HPVL cattle. These results suggest that alteration of hsa-miR-424-5p in milk sEVs are associated with BLV progression to onset of EBL. This study also indicates that hsa-miR-424-5p in milk sEVs could be a non-invasive biomarker for identifying cattle at risk of developing EBL.

## 1. Introduction

Enzootic bovine leukosis (EBL) is a malignant B-cell lymphosarcoma caused by the oncogenic bovine leukemia virus (BLV). BLV belongs to the genus *Deltaretrovirus* in the family *Retroviridae* and is closely related to human T-lymphotropic virus types 1 and 2 [1]. According to the World Organization for Animal Health (WOAH), EBL is a notifiable disease in many countries and is currently one of the most commonly reported neoplastic diseases in cattle [2]. BLV can be transmitted between animals through infected biological materials, such as blood, saliva, and semen, and may also pass from an infected dam to the fetus during pregnancy via the placenta [3,4]. Transmission of BLV can also occur during routine veterinary procedures including blood collection and rectal examination, particularly when syringe needles and disposable rectal gloves are reused between BLV-infected and susceptible cattle [3,4]. In addition, blood-sucking insects such as tabanid flies (*Tabanus* spp.) and stable flies (*Stomoxys calcitrans*) play important roles in the mechanical transmission of BLV between cattle [5,6]. BLV infection is characterized by the iceberg principle, in which 70% of the infected cattle do not show any clinical signs of the disease [7]. Approximately 30% of BLV-infected cattle develop a benign condition, termed persistent lymphocytosis, which is characterized by a permanent increase in the number of circulating B lymphocytes. Only a small percentage of BLV-infected cattle (approximately 1–5%) develop EBL, which is fatal [6]. BLV-infected cattle suspected of developing EBL have been reported to show lethargy, loss of appetite, weight loss, fever, enlargement of lymph node, protrusion of the conjunctival membrane, bulging of eye, reduced reproductive efficiency, and decreased milk production [8]. Moreover, BLV causes substantial direct and indirect economic losses to the dairy industry, including increased replacement costs, reduced fertility and productivity, carcass condemnation, and restrictions on the export of live cattle, semen, and embryos to countries with BLV control programs [9].

Although BLV infection occurs worldwide, several countries have successfully eradicated BLV infection, including Belgium, Denmark, Germany, Finland, The Netherlands, and the UK, by implementing a nationwide survey and slaughter of BLV-infected cattle [10,11,12]. Previous studies have reported that the prevalence of BLV infection is high in some countries, including the USA, China, and Japan [13,14]. In Japan, a nationwide serological survey revealed that the prevalence of antibodies against BLV was 40.9% in dairy cattle and 28.7% in beef-breeding cattle [15]. Importantly, a recent governmental report showed that the number of cattle diagnosed with EBL at farms and slaughterhouses has increased gradually every year (https://www.maff.go.jp/j/syouan/douei/kansi_densen/attach/pdf/kansi_densen-22.pdf, accessed on 10 January 2026). In Japan, milk, meat, and other relevant products from cattle diagnosed with EBL are not legally eligible for human consumption because of a policy that requires meat and milk products to be supplied only from healthy cattle [16]. To mitigate economic losses and control disease spread, early prognosis of EBL in cattle and prevention of BLV transmission to healthy cattle are highly preferred. Several diagnostic methods are available for the detection of BLV including agar gel immunodiffusion, radioimmunoassay, enzyme-linked immunosorbent assay, conventional polymerase chain reaction PCR and quantitative real-time PCR (qPCR) [17,18]. However, a recent study reported that over half of the EBL cattle were detected via postmortem inspections at slaughterhouses, not during on-farm inspections [19]. This indicates that the existing diagnostic methods may be insufficient for the early detection of EBL in cattle.

Previous studies have reported that the alterations in serum lactate dehydrogenase (LDH) activity and its isoenzymes ratio LDH 2 and 3 (LDH 2% and LDH 3%) increased in cattle affected by EBL [20]. More recently, LDH 2% and total LDH have been evaluated as potential biomarkers for EBL, with significantly higher levels observed in EBL cattle compared with non-EBL cattle [21]. However, LDH is not specific to EBL and may also be influenced by other pathological conditions and host factors, limiting its prognostic specificity [21]. Nevertheless, LDH may provide useful biochemical information on cellular or tissue alterations associated with BLV infection and EBL progression. Therefore, evaluating LDH together with molecular and hematological parameters may provide additional insight into the biological changes associated with BLV infection and disease progression. A previous study reported that BLV-infected cattle with a high proviral load (HPVL) (≥10,000 DNA copies/10^5^ white blood cells [WBCs]) are at higher risk of developing EBL compared with low-proviral-load (LPVL) (<10,000 DNA copies/10^5^ WBCs) cattle [22]. BLV proviral DNA represents the integrated DNA form of the virus within the genome of BLV-infected host cells and persists in BLV-infected lymphocytes. The quantification of BLV proviral DNA is expressed as proviral load [23]. It provides an estimate of the burden of BLV-infected cells and is therefore useful for assessing infection status and the risk of disease progression. However, it remains difficult to predict which high-risk cattle will subsequently progress to EBL based solely on proviral load. Moreover, EBL has also been reported in a limited number of cattle with LPVL [24]. This discrepancy suggests that proviral load is necessary but not sufficient to explain accurately about EBL progression. Consequently, the biological mechanisms driving EBL development in cattle from LPVL to HPVL remain largely unresolved. Therefore, for a more accurate identification, it is essential to focus on host factors, as well as cellular or molecular mechanisms, to identify reliable biomarkers associated with disease progression.

Extracellular vesicles (EVs) are membranous structures that are found in many biological fluids including serum, plasma, and milk [25,26]. According to the Minimal Information for Studies of Extracellular Vesicles (MISEV2023) guidelines, there are different classes of EVs, including small and large EVs [27]. Moreover, the use of the term “small EVs” (sEVs) (<200 nm in diameter) instead of “exosomes” was recommended. These sEVs contain mRNA, microRNAs (miRNAs), DNA, proteins, and lipids that facilitate intercellular communication [28,29]. In human breast cancer, sEVs released by fibroblasts are selectively taken up by cancer cells to promote their migration, activity, and metastasis [30]. miRNAs are endogenous small non-coding RNAs that target mRNAs for translational control [31]. A previous study reported that miRNAs such as miR-223 and miR-142-5p were significantly upregulated in milk sEVs from mastitis-infected cattle, and these miRNAs could be considered potential biomarker candidates for mastitis [32]. Another study reported that the abundance of bta-miR-424-5p in milk sEVs was significantly higher in BLV-infected cattle with HPVL compared with Control cattle [33]. Moreover, a recent study detected increased level of bta-miR-424-5p in milk sEVs from cattle diagnosed with EBL [34]. These findings suggested that hsa-miR-424-5p plays an important role in BLV-associated pathogenesis and progression to the onset of EBL. However, despite these observations, the temporal dynamics and regulatory patterns of hsa-miR-424-5p across the different clinical stages of BLV infection remain poorly understood, particularly during the transition from early infection to EBL onset. Understanding the molecular profile of hsa-miR-424-5p throughout disease progression is essential to determine its potential as a predictive biomarker for identifying cattle at risk of developing EBL and for understanding the underlying mechanisms of disease progression. Therefore, this study aimed to investigate the pattern of hsa-miR-424-5p in milk sEVs across the clinical stages of BLV infection. By evaluating the stage-specific changes in hsa-miR-424-5p, this study sought to clarify whether alterations in its levels could serve as a reliable molecular indicator for predicting the onset of EBL. Our findings may contribute to the development of non-invasive prognostic strategies for monitoring BLV disease progression and improving herd-level management and control programs in dairy cattle.

## 2. Materials and Methods

### 2.1. Hematological Examination

Blood samples were collected from BLV-uninfected healthy cattle (Control) as well as BLV-infected cattle with LPVL (<10,000 DNA copies/10^5^ WBCs), HPVL (≥10,000 DNA copies/10^5^ WBCs), and EBL from dairy farms and a slaughterhouse located in various places in Japan (Table S1). The identification of cattle with clinical EBL was based on the findings obtained from both ante-mortem and postmortem examinations [21]. During the ante-mortem examination, cattle were assessed for clinical findings consistent with EBL including emaciation, enlargement of superficial lymph nodes, exophthalmos (protrusion of the eye), increased peripheral blood lymphocyte counts (≥12,000 cells/mm^3^), and/or the presence of neoplastic B cells in peripheral blood. During the postmortem examination, characteristic findings included generalized enlargement of lymph nodes and the presence of tumor masses or diffuse lymphomatous infiltration in visceral organs, particularly the heart, abomasum, kidneys, liver, spleen, and lungs. These gross pathological findings, along with the ante-mortem clinical and hematological examination, were used to identify EBL cattle. Blood samples obtained from the cattle were immediately aliquoted into vacuum blood collection tubes with or without heparin (VENOJECT II VP-H070K or VP-AS076K; Terumo, Tokyo, Japan) for subsequent analyses. Total WBC and lymphocyte counts were measured using a cell counter (Celltac α MEK-6550; Nihon Kohden, Tokyo, Japan).

### 2.2. Detection of BLV Provirus

Plasma was removed from 1.0 mL of the anticoagulated blood via centrifugation at 587× *g* for 15 min at RT in a TMA-29 rotor using an MX-307 centrifuge (Tomy, Tokyo, Japan). For hemolysis, 1.0 mL of 0.83% NH_4_Cl and 0.01% EDTA in distilled water (DW) were added to the pelleted hemocytes and vortexed, followed by incubation for 5 min at RT. After hemolysis, WBCs were separated via centrifugation at 587× *g* for 5 min at RT in a TMA-29 rotor using an MX-307 centrifuge. The supernatant was discarded and phosphate-buffered saline (PBS) was added to wash the WBCs. The washed WBCs were then separated via centrifugation at 587× *g* for 5 min at RT, and the supernatant was discarded. The separated WBCs were suspended in 200 µL of PBS. Total DNA was extracted from the WBCs using a DNeasy Blood & Tissue Kit (69506; Qiagen, Hilden, Germany). Nested-PCR was then performed to detect the BLV pX with an amplicon size of 280 bp [35] or the envelope region with an amplicon size of 444 bp [36] using a GoTaq Hot Start Green Master Mix (M512C; Promega, Madison, WI, USA). The thermal cycling conditions were: 94 °C for 2 min, 25 cycles of 94 °C for 45 s for denaturation, 62 °C for 30 s for annealing, 72 °C for 30 s for amplification, and 72 °C for 4 min for final extension. Finally, the PCR products were electrophoresed on a 1% agarose gel and visualized using ethidium bromide staining.

### 2.3. Measurement of BLV Proviral Load

The extracted DNA was also used to measure the BLV proviral load (copies/10^5^ WBCs) via quantitative real-time PCR (qPCR) with a CoCoMo-BLV Primer/Probe (A803; RIKEN Genesis, Tokyo, Japan), according to the manufacturer’s instructions. Hematological tests, detection of serum antibodies against BLV, and measurement of BLV copy number were conducted by the Gifu Prefectural Chuo Livestock Hygiene Service Center (Gifu, Japan).

### 2.4. Analysis of Lactate Dehydrogenases (LDH) Levels

LDH isozymes in the serum or plasma were measured with a Hydrasys 2 Scan system (Sebia, Paris, France) using HYDRAGEL 7 ISO-LDH (Sebia), which was conducted by the clinical laboratory testing company SRL (Tokyo, Japan).

### 2.5. Isolation and Characterization of sEVs

Raw milk samples (100 mL) were collected from dairy cattle and centrifuged at 2000× *g* for 20 min at 4 °C in an A2506 rotor using a model 7000 centrifuge (Kubota, Tokyo, Japan) to remove milk fat globules, somatic cells, and cell debris according to previous reports with slight modifications [37,38]. Briefly, defatted milk samples were warmed for 10 min at 37 °C, and then acetic acid (Wako, Osaka, Japan) was added [milk/acetic acid = 100 (*v*/*v*)], followed by stirring and incubation to generate casein aggregates. To remove casein, milk samples were centrifuged at 5000× *g* for 20 min at RT. The supernatant (whey) was filtrated sequentially through 1.0, 0.45, and 0.2 µm pore size filters (GA-100, C045A047A, and C020A047A, respectively, Advantec, Tokyo, Japan).

Milk sEV in whey was characterized using transmission electron microscopy (TEM), nanoparticle tracking analysis (NTA), and Western blotting (WB) according to the MISEV2023 guidelines, as described previously with slight modifications [39,40]. For TEM observation, whey was ultracentrifuged at 100,000× *g* for 1 h at 4 °C in a P40ST swing rotor (Hitachi Koki, Tokyo, Japan) using a Himac CP80NX ultracentrifuge (Hitachi Koki). The pellets were suspended in 1 mL of PBS and layered on the top of a linear sucrose density gradient (SDG) solution (3 mL each of 10−20−40% sucrose in DW [*w*/*v*]), and ultracentrifuged at 200,000× *g* for 18 h at 4 °C in a P40ST swing rotor. Then, 0.9 mL of each gradient fraction was collected from the top of the tube and numbered 1–12. Fraction no. 12 of the SDG solution was collected for sEVs recovery as this fraction corresponds to the milk sEVs enriched fraction as described previously [33]. The sucrose density gradient fraction no.12 was diluted with 10 mL of 0.1 µm-filtered DW and ultracentrifuged again at 100,000× *g* for 1 h at 4 °C in a P40ST swing rotor. The sEVs pellets were suspended in 100 µL of 0.1 µm-filtered DW and collected in another tube. Milk sEVs suspension was diluted at 1:50 with 0.1 µm-filtered DW and applied onto glow-discharged polyvinyl butyral support films on copper grids (U1013; EM Japan, Tokyo, Japan). The grids were stained with uranyl acetate, and the excess solution was removed using a filter paper. The dried grids were examined using a JEM-2100F electron microscope (JEOL, Tokyo, Japan) at 200 kV.

For NTA, the sEVs suspension was diluted 1:100 with 0.1 µm-filtered DW, followed by filtration with a 0.22 µm filter. The nanoparticle size distribution was analyzed using a Zetasizer Nano ZS nanoparticle analyzer (Malvern Panalytical, Worcestershire, UK).

For WB analysis, the proteins from the sEVs suspension were transferred to polyvinylidene difluoride (PVDF) blotting membranes and blocked with 5% skim milk in Tris-buffered saline (0.1 M Tris-HCl [pH 8.0] and 0.03 M NaCl) containing 0.1% Tween-20 (TBST) at RT for 30 min. The membrane was then incubated with primary antibodies against the milk sEVs surface marker protein CD63 (1:400, sc-31214, Santa Cruz Biotechnology, Dallas, TX, USA) and the internal protein HSP70 (1:100, N27F3-4, Enzo Life Science, Farmingdale, NY, USA) diluted in 1% skim milk in TBST for 1 h at RT. After washing three times with TBST, the membrane was incubated with donkey anti-goat F(ab′)_2_-HRP (1:2000, sc-3851, Santa Cruz Biotechnology) or anti-mouse IgG, with HRP-linked antibody (1:2000, #7076BC, Cell Signaling Technology, Danvers, MA, USA) diluted in 1% skim milk in TBST for 1 h at RT. After washing three times, peroxidase activity was detected using a Pierce ECL Plus Western Blotting Substrate (32132, Thermo Fisher Scientific, Waltham, MA, USA) and visualized using ChemiDoc XPS+ (Bio-Rad Laboratories, Hercules, CA, USA).

### 2.6. RNA Extraction and cDNA Synthesis

Whey was ultracentrifuged in two successive ultracentrifugation steps at 100,000× *g* for 1 h at 4 °C in a P42A angle rotor (Hitachi Koki) using a Himac CP80NX ultracentrifuge. After the first ultracentrifugation, the supernatant was discarded, and the pellets were suspended in 1 mL of DW and collected in new 13 PET tubes (Eppendorf Himac Technologies, Hitachinaka, Japan). After that, 8 mL of DW was added, and the mixture was ultracentrifuged at 100,000× *g* for 1 h at 4 °C in a P40ST swing rotor. RNA was extracted from the collected milk sEVs pellets using the miRNeasy Serum and Plasma Kit (217184; Qiagen). The concentration of the extracted RNA was determined using a QuantiFluor RNA System (E3310, Promega). cDNA was synthesized from 10 ng of the extracted total RNA using a miRCURY LNA RT Kit (339340; Qiagen) according to the manufacturer’s instructions. The mixture was then reverse-transcribed in 10 µL of a reaction solution and incubated for 60 min at 42 °C, followed by incubation for 5 min at 95 °C to inactivate the reverse transcription enzyme.

### 2.7. Validation of Hsa-miR-424-5p Level Using qPCR

The primers used for the validation of hsa-miR-424-5p (YP00204736, Qiagen) were included in the miRCURY LNA miRNA PCR Primer Assay components (339306; Qiagen), as previously reported [33,34]. For qPCR analysis, cattle were classified into four groups such as Control cattle, LPVL (<10,000 BLV proviral DNA copies/10^5^ WBCs), HPVL (≥10,000 copies/10^5^ WBCs), and EBL cattle according to the previous reports [22,39,41]. Although total WBC counts and lymphocyte percentages were measured, persistent lymphocytosis was not independently classified because hematological measurements were obtained at a single time point and longitudinal assessment is required to establish persistence. Prior to qPCR analysis, the cDNA was diluted 1:30 by adding RNase-free water, and 3.0 μL of diluted cDNA was used in a total reaction volume of 10 μL using a StepOne Plus analytical thermal cycler (Applied Biosystems, Foster City, CA, USA). The thermal cycling program was as follows: 95 °C for 2 min for initial heat activation, followed by 40 cycles at 95 °C for 10 s for denaturation and 56 °C for 1 min for annealing and extension. After amplification, a melting curve analysis was performed to validate the specificity of the reactions. The miRNA content was compared by calculating the threshold cycle (Ct) values.

### 2.8. Statistical Analysis

Statistical analysis was performed using the Mann–Whitney U test, and the correlation coefficients were analyzed using Spearman’s correlation. All data visualizations were performed in Python ver. 3.14.6 using Matplotlib ver. 3.10.5 [42] and Seaborn ver. 0.13.2 [43]. Results were considered statistically significant when the *p*-values were less than 0.05.

## 3. Results

### 3.1. Clinical Status of BLV Infection

The hematological data of the cattle used in this study are summarized in Table S1. A total of 159 Holstein Frisian cattle aged 27–172 months were included in this study, which were categorized into four groups: BLV-uninfected healthy cattle (Control) (*n* = 45), LPVL cattle (*n* = 28), HPVL cattle (*n* = 46), and EBL cattle (*n* = 40). These findings indicate a progressive shift in hematological and biochemical parameters corresponding to disease severity from LPVL to EBL in cattle.

### 3.2. Characterization of Milk sEVs

The morphology of the isolated milk sEVs was observed using TEM, which showed a round shape with diameters below 200 nm (Figure 1A). NTA showed that the mean peak size distribution was <200 nm (Figure 1B). The milk sEV surface marker protein CD63 and the internal protein HSP70 were successfully detected via WB analysis (Figure 1C and Figure S1). These results indicate the successful isolation and characterization of milk sEVs.

### 3.3. Validation of Hsa-miR-424-5p Level by qPCR

The qPCR results representing the distribution of Ct values of hsa-miR-424-5p in milk sEVs across individual cattle samples are shown in Figure 2 and Figure 3. The Ct values ranged broadly among all the samples and were arranged in descending order to visualize the overall distribution across the clinical stages of the different groups. The results indicate that the Ct value of hsa-miR-424-5p was consistently detectable in milk sEVs from the Control cattle and BLV-infected cattle with LPVL and HPVL until the onset of EBL. This overall distribution pattern suggests that hsa-miR-424-5p is broadly encapsulated in milk sEVs and can be reliably detected across the different clinical stages of BLV infection. The distribution of the Ct values of hsa-miR-424-5p in LPVL and HPVL cattle was generally higher than that in Control cattle; however, it was not significantly different (*p* > 0.05). The increase in Ct values indicated that the abundance of hsa-miR-424-5p in milk sEVs from LPVL and HPVL cattle was lower than that in Control cattle. In contrast, EBL cattle showed relatively lower Ct values, indicating higher levels of hsa-miR-424-5p in milk sEVs than in Control and HPVL cattle. Statistical analysis revealed significant differences between the Control vs. EBL (*p* < 0.05) and HPVL vs. EBL groups (*p* < 0.05). These results suggest that the Ct values of hsa-miR-424-5p in milk sEVs largely fluctuated throughout BLV infection and differed significantly during the onset of EBL.

### 3.4. Correlation Analysis

To evaluate the potential association of hsa-miR-424-5p with clinical and hematological parameters related to the clinical stages of BLV infection and the onset of EBL, the Ct values obtained from qPCR analysis were assessed using Spearman’s correlation analysis with several variables, including age, WBC count, lymphocyte count, BLV copy number, total LDH activity, and LDH isozyme 2 + 3% (Figure 4 and Figure S2). The results demonstrated a statistically significant negative correlation between hsa-miR-424-5p and LDH 2 + 3% (r = −0.28, *p* < 0.001), indicating that higher miRNA abundance was associated with lower LDH 2 + 3% activity. In contrast, no statistically significant correlations were observed between hsa-miR-424-5p and the remaining clinical variables, including age (r = −0.00), WBCs (r = 0.11), lymphocyte count (Ly; r = 0.07), BLV copy (r = 0.01), or total LDH activity (r = −0.11). These findings suggest that hsa-miR-424-5p abundance is largely independent of general hematological indices and viral load yet exhibits a specific and significant inverse association with LDH 2 + 3%, which may reflect a functional relationship between this miRNA and the LDH isoform ratio in the context of BLV-associated disease progression.

### 3.5. Receiver Operating Characteristic (ROC) Curve Analysis

ROC curve analysis was performed to evaluate the prognostic performance of hsa-miR-424-5p in milk sEVs to distinguish different stages of BLV infection from the onset of EBL (Figure 5). The area under the curve (AUC) was used as the primary measure of classification accuracy of the ROC curve analysis results. The results showed that the BLV vs. EBL comparison yielded the highest AUC value (0.71), indicating a moderate discriminative capacity between the two clinical stages. The Control vs. BLV comparison demonstrated an AUC of 0.63, reflecting a fair ability to differentiate healthy Control from BLV-affected cattle. Similarly, the Control vs. EBL comparison produced an AUC of 0.64 (reverse), suggesting a fair but comparatively fluctuating separation between the Control and EBL cattle. The Control vs. all comparisons exhibited moderate discriminative performance (AUC = 0.54), approaching near-chance level classification when the Control cattle were compared against BLV infection at the onset of EBL. Notably, the reverse curves observed for the Control vs. EBL and BLV vs. EBL comparisons indicated an inverse relationship between the predictor variable and the probability of disease stage, implying that moderate-to-high biomarker values were associated with a different phenomenon of disease progression. Collectively, these findings suggest that while the model demonstrates statistically meaningful discrimination across the clinical stages of BLV infection to the onset of EBL, its performance is the most robust in distinguishing BLV infection from the onset of EBL.

## 4. Discussion

Several studies in humans have demonstrated that miRNAs play critical roles in the pathogenesis of cancer, functioning as oncogenic agents that promote tumor initiation and progression [44,45]. Circulating miRNAs in the blood have been studied as biomarkers for monitoring various cancers in humans [46,47]. However, blood collection from cattle requires veterinary expertise and is inherently invasive, potentially causing stress and discomfort to the animals. In contrast, milk collection can be performed routinely by farmers in a non-invasive manner, allowing frequent sampling. In the current study, the distinctive patterns of hsa-miR-424-5p across the clinical stages of BLV infection to the development of EBL represent important insights into its stage-specific alterations during disease progression. Importantly, in the present study, LPVL and HPVL cattle showed relatively higher Ct values than Control cattle, suggesting a reduction in hsa-miR-424-5p abundance during the preclinical stages of BLV infection. In contrast, EBL cattle had significantly lower Ct values (*p* < 0.05), reflecting a marked increase in hsa-miR-424-5p abundance at the clinical stage of disease onset. This distinct shift from reduced abundance in LPVL and HPVL cattle to elevated abundance in EBL cattle suggests the stage-specific regulation of hsa-miR-424-5p. It implies that hsa-miR-424-5p is not directly associated with viral replication dynamics, but rather with cellular changes in the host accompanying malignant progression [48]. Therefore, these findings support the notion that hsa-miR-424-5p may serve as a biomarker of early stage of EBL development rather than as a predictive indicator during the early stages of BLV infection.

Our results are consistent with previous findings that demonstrated significantly elevated levels of hsa-miR-424-5p in milk sEVs from EBL cattle compared to those in Control cattle [34]. This study identified hsa-miR-424-5p as a biomarker associated with EBL onset and suggests its involvement in oncogenic pathways. However, Tsukada et al. [34] did not include LPVL and HPVL cattle during qPCR validation, therefore limiting the assessment of the temporal dynamics of EBL disease progression. In contrast, Nakanishi et al. [33] showed increased levels of hsa-miR-424-5p in BLV-infected cattle compared with Control cattle. However, their qPCR validation was limited to HPVL cattle only, without the inclusion of LPVL cattle and EBL cattle. As a result, the selective differences in the study design of these studies may have biased the findings toward HPVL cattle. Moreover, it did not fully capture the spectrum of the clinical stages of BLV infection. Additionally, the relatively small sample sizes of previous studies may have limited the robustness of our conclusions. By incorporating both LPVL and HPVL cattle, the present study provides a more comprehensive evaluation of disease progression. The findings also demonstrate that the levels of hsa-miR-424-5p show a gradual increase in abundance with disease progression, thereby refining its prognostic interpretation.

A previous study showed that the levels of several miRNAs, including bta-miR-24-3p, were significantly elevated in blood sEVs from EBL cattle compared with those from Control cattle [49]. Therefore, the findings suggest their potential utility as prognostic biomarkers in blood [49]. However, our current study findings are not consistent with that result, which may be attributed to differences in the biological sample types obtained. While Takada et al. [49] focused on blood sEVs, our study investigated hsa-miR-424-5p in milk sEVs, which may reflect the distinct physiological and pathological processes associated with BLV infection. In contrast, another study demonstrated that bta-miR-375 in the serum is a potential biomarker for the early diagnosis of EBL in cattle [50]. While this previous study utilized serum samples without sEVs, our study focused on milk sEVs, which represent a distinct perspective of BLV pathogenesis. Furthermore, sEV-derived miRNAs are encapsulated within sEVs, potentially leading to differences in disease dynamics compared to circulating free miRNAs in the serum. This distinction highlights the complementary nature of different miRNA sources [51]. Therefore, our findings indicate that hsa-miR-424-5p in milk sEVs may serve as a prognostic biomarker of EBL, reflecting its unique patho-physiological processes.

A previous study has shown that hsa-miR-424-5p acts as an oncogene that promotes the proliferation and metastasis of colorectal cancer in humans by directly targeting and inhibiting the expression of *SCN4B* [52]. Another report implicated hsa-miR-424-5p in multiple oncogenic pathways in humans, including cell cycle regulation, PI3K/AKT signaling, and modulation of tumor suppressor genes [53,54]. These findings indicate that hsa-miR-424-5p functions as an important regulatory molecule in tumorigenesis and may serve as a potential biomarker or therapeutic target in cancer-related studies. Importantly, these molecular functions are highly relevant to the clinical progression of BLV leukemogenesis, in which infected B cells undergo clonal expansion, evade host immune surveillance, and ultimately progress toward EBL onset. These findings suggest a possible mechanistic role of hsa-miR-424-5p in disease progression. Therefore, the elevated levels of hsa-miR-424-5p observed in EBL cattle may reflect the increased secretion of oncogenic miRNAs from transformed B cells, potentially contributing to tumor progression and systemic signaling. This is consistent with previous reports suggesting that sEVs-associated miRNAs in blood can serve as biomarkers for cancer detection in humans [55,56]. This dual role further suggests that its abundance is not simply driven by viral infection or proviral load, but rather reflects complex host–pathogen interactions and tumor-specific regulatory networks. In the clinical stage of BLV infection, the lack of hsa-miR-424-5p upregulation in LPVL and HPVL cattle suggests that its dysregulation may not be involved in early viral replication or latency. In contrast, the increased abundance observed in EBL cattle may indicate that dysregulation of hsa-miR-424-5p is more closely associated with malignant transformation.

BLV and HTLV-1 or HTLV-2 belong to the same genus, sharing similar biological characteristics including infection of lymphoid cells and association with lymphoproliferation [1]. Therefore, findings on miRNA alterations in HTLV infection may provide a relevant comparative context for BLV-associated disease. Although hsa-miR-424-5p has not been reported in HTLV-1 or HTLV-2 infection, several other miRNAs have been identified in HTLV-associated tissues, peripheral blood, and sEVs. These findings support the role of miRNA-mediated regulatory networks in retroviral persistence, modulation of host immune responses, and disease progression, including leukemogenesis. A recent study identified several miRNAs including miR-320c, miR-629-5p, and miR-944 in sEVs from HTLV-1-infected HuT102 cells, suggesting that these miRNAs may regulate viral transmission and host–virus interactions [57]. Another study identified hsa-miR-20b-5p and hsa-let-7e-5p in blood from HTLV-1/2-infected human patients, suggesting that dysregulated miRNAs may contribute to HTLV pathogenesis and immune responses [58]. Therefore, the identification of hsa-miR-424-5p expands the repertoire of miRNAs potentially associated with *deltaretrovirus* infection and warrants further functional investigation. The expression of has-miR-424-5p was significantly downregulated in hepatocellular carcinoma (HCC) while restoration of hsa-miR-424-5p suppressed malignant cellular behaviors and inhibited tumor growth in vivo [59]. Moreover, hsa-miR-424-5p overexpression is significantly associated with distant metastasis in human thyroid cancer [60]. In contrast, decreased levels of has-miR-424-5p in human breast cancer are associated with advanced clinical stages, higher metastatic lymph node count, and lower histological grade [61]. Zhou et al. [62] demonstrated that sEVs-encapsulated hsa-miR-424-5p inhibited PD-L1 signaling in triple-negative breast cancer, consequently promoting tumor cell apoptosis in vitro and inhibiting tumor growth in vivo. These phenomena suggest that sEVs-delivered hsa-miR-424-5p is a promising immune checkpoint blockade strategy with broad implications for tumor immunotherapy in humans. In addition to its role in cancer, hsa-miR-424-5p exacerbates insulin resistance progression in human liver cells and may serve as a potential biomarker for metabolic syndrome [63].

Our study did not identify a correlation between hsa-miR-424-5p and different hematological variables, except for LDH 2 + 3%, which showed a statistically significant negative correlation with hsa-miR-424-5p levels. LDH is a cytoplasmic enzyme released into the circulation upon cell death and is well-recognized as a surrogate marker of cellular injury and tissue destruction. Elevated serum LDH activity is commonly observed in hematological malignancies, including EBL [40,64]. Notably, total LDH activity in the blood has been reported to increase in EBL-affected cattle and LDH 2 + 3% isozymes have been identified as diagnostically useful markers for EBL [64]. Furthermore, an increased ratio of LDH 2 + 3% isozymes is clinically associated with hematological malignancies, including leukemia [65]. These phenomena reflect enhanced cell turnover and metabolic reprogramming during malignant progression. A previous study reported that serum levels of LDH 2 + 3% isozymes were elevated in EBL cattle compared to non-EBL cattle, reflecting increased cellular alterations and tumor burden during disease progression [20]. The significant inverse association observed specifically with LDH 2 + 3% isozymes suggests that the higher abundance of hsa-miR-424-5p is not attributable to generalized cellular injury or nonspecific cell lysis. Rather, it may reflect a disease stage-specific biological phenomenon associated with the lymphoproliferative process characteristic of EBL progression. Therefore, these findings imply that the abundance of sEVs-encapsulated hsa-miR-424-5p is not passively influenced by cell-injury-driven release mechanisms, but may instead represent an actively regulated molecular response to BLV-associated oncogenic transformation to EBL. When considered alongside previous studies, these results provide further insight into the mechanisms underlying EBL progression. Nevertheless, additional studies involving larger, independent cohorts and functional validation are required to establish the clinical utility of hsa-miR-424-5p biomarkers and to determine their potential application in predicting EBL onset, improving early prognosis, and guiding the management of BLV-infected cattle.

The current study has several limitations. First, our study used the commercial miRCURY LNA PCR assay for miR-424-5p quantification that was designed for hsa-miR-424-5p and may detect both hsa-miR-424-5p and bta-miR-424-5p because their mature sequences differ by only one nucleotide at the 3′ end. Therefore, the present qPCR assay cannot unambiguously discriminate between the human and bovine homologues in bovine milk samples. Accordingly, the detected signal should be interpreted as miR-424-5p rather than as definitive species-specific detection of hsa-miR-424-5p or bta-miR-424-5p. Future studies using sequence-specific assays or direct sequencing of the amplified products will be necessary to definitively determine the species origin of miR-424-5p in bovine milk. Second, the study lacks a validated endogenous miRNA reference for normalization of miRNA abundance in bovine milk sEVs. Notably, a miRNA that is stably encapsulated in milk sEVs and remains unaffected by BLV infection status would provide an appropriate normalization control. Previous studies from our group therefore standardized the amount of extracted RNA used for reverse transcription rather than relying on an unvalidated endogenous miRNA reference [33,34]. Although the same approach was used in the present study, this may not completely account for biological and technical variations in sEVs-associated miRNA abundance. Future studies should systematically evaluate candidate sEVs-associated reference miRNAs across BLV-infected and Control cattle and validate their stability before using them for normalization. Beyond the study limitation, our results highlight the biological mechanism underlying sEVs-associated miR-424-5p dynamics from LPVL to HPVL and the onset of EBL and underscore the limitations of single-biomarker approaches for disease stratification. Future investigations should prioritize integrative multi-omics frameworks to identify composite biomarker signatures with improved sensitivity and specificity for the early detection and risk stratification of EBL.

## 5. Conclusions

Predicting which BLV-infected cattle will develop EBL is currently not possible and unclear. However, the current study provides evidence that hsa-miR-424-5p in milk sEVs shows stage-dependent modulation during BLV infection, with a marked increase and abundance observed in EBL cattle. Despite this association, hsa-miR-424-5p demonstrated no correlation with the BLV proviral load, host age, or other hematological indicators. However, a strong negative correlation was observed between hsa-miR-424-5p abundance and LDH 2 + 3% levels. Moreover, the ROC results suggested that hsa-miR-424-5p has moderate but clinically relevant prognostic potential for discriminating between BLV-infected cattle and EBL cattle. These findings indicate that hsa-miR-424-5p adequately captures the full clinical stages of BLV progression to the onset of EBL. The use of milk as a non-invasive and readily accessible biological matrix supports its applicability for the longitudinal monitoring of BLV-infected cattle under field conditions.

## Figures and Tables

**Figure 1:**
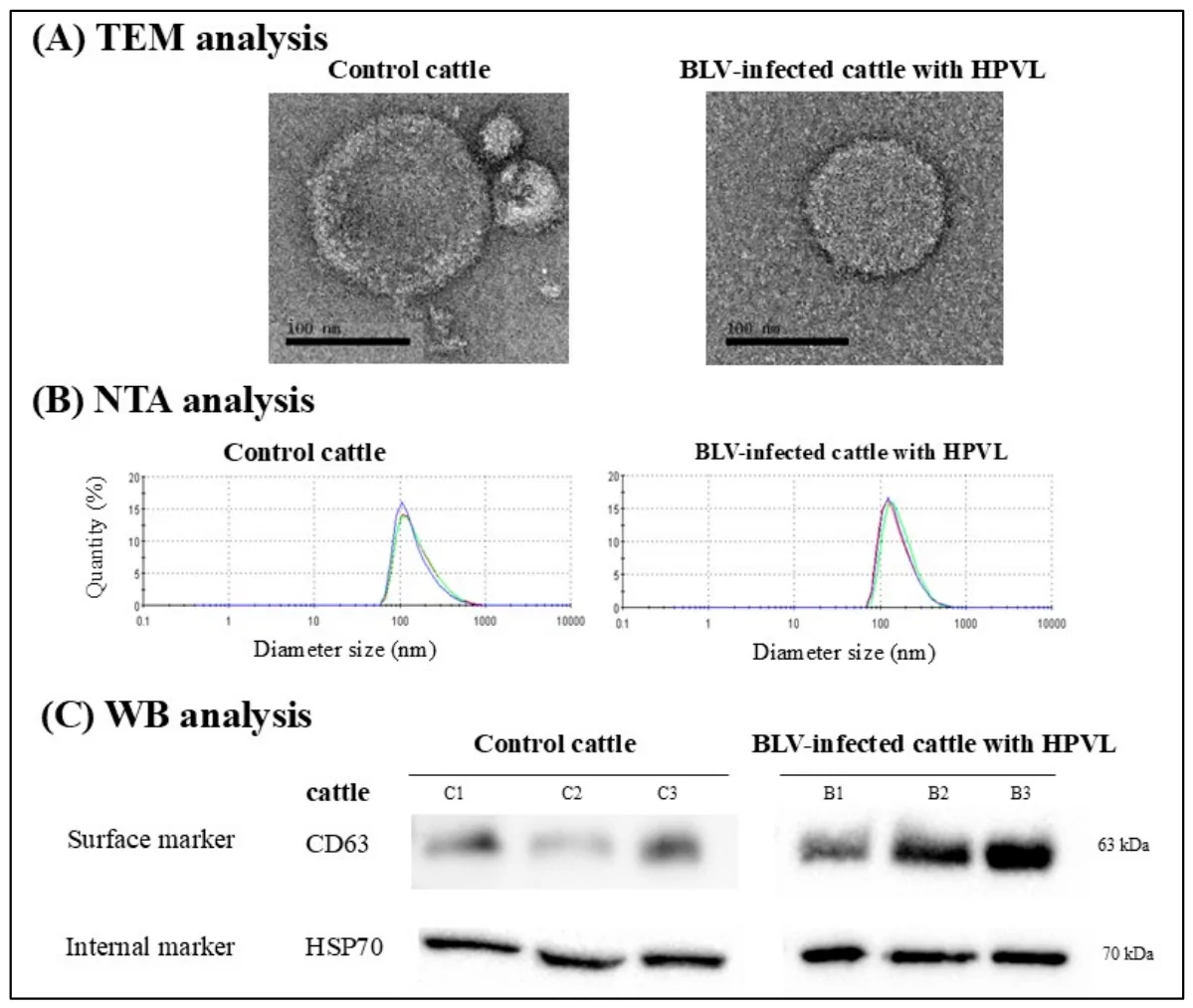
Characterization of milk small extracellular vesicles (sEVs). (**A**) Transmission electron microscopy (TEM) analysis of milk sEVs. Representative TEM images showing the morphology of sEVs isolated from Control (C1–C3) and BLV-infected cattle (B1–B3) with high proviral load (HPVL). The sEVs images show the characteristic cup-shaped morphology with a lipid bilayer membrane. Scale bars are shown in the respective panels. (**B**) Representative nanoparticle tracking analysis (NTA) profiles showing the size distribution of milk sEVs isolated from three Control and three BLV-infected cattle with HPVL. (**C**) Representative Western blot (WB) images confirming the presence of sEVs marker proteins, including CD63 and HSP70, in three Control and three BLV-infected cattle with HPVL, indicating the successful isolation of sEVs. B, BLV-infected cattle; C, Control cattle.

**Figure 2:**
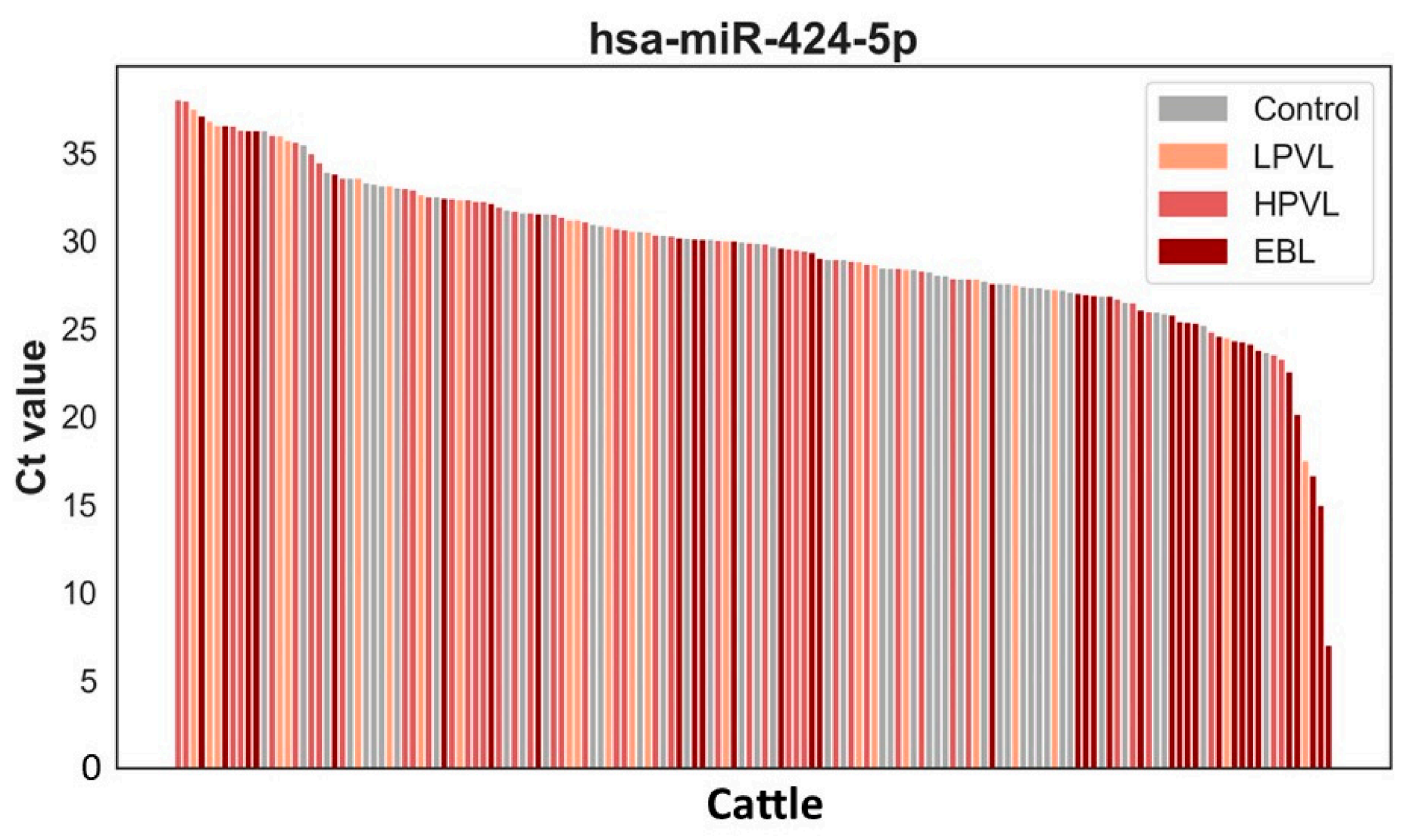
Waterfall plot of hsa-miR-424-5p in milk small extracellular vesicles (sEVs). Waterfall plot showing individual qPCR cycle threshold (Ct) values of hsa-miR-424-5p for all 159 samples, ranked from the highest to lowest. Each vertical bar represents a single sample and is color-coded according to group: Control (gray), low proviral load (LPVL) (light orange), high proviral load (HPVL) (salmon), and enzootic bovine leukosis (EBL) (dark red). The plot illustrates the continuous distribution, inter-individual variability, and overlap of Ct values among groups. Lower Ct values correspond to higher relative abundance of hsa-miR-424-5p in milk sEVs.

**Figure 3:**
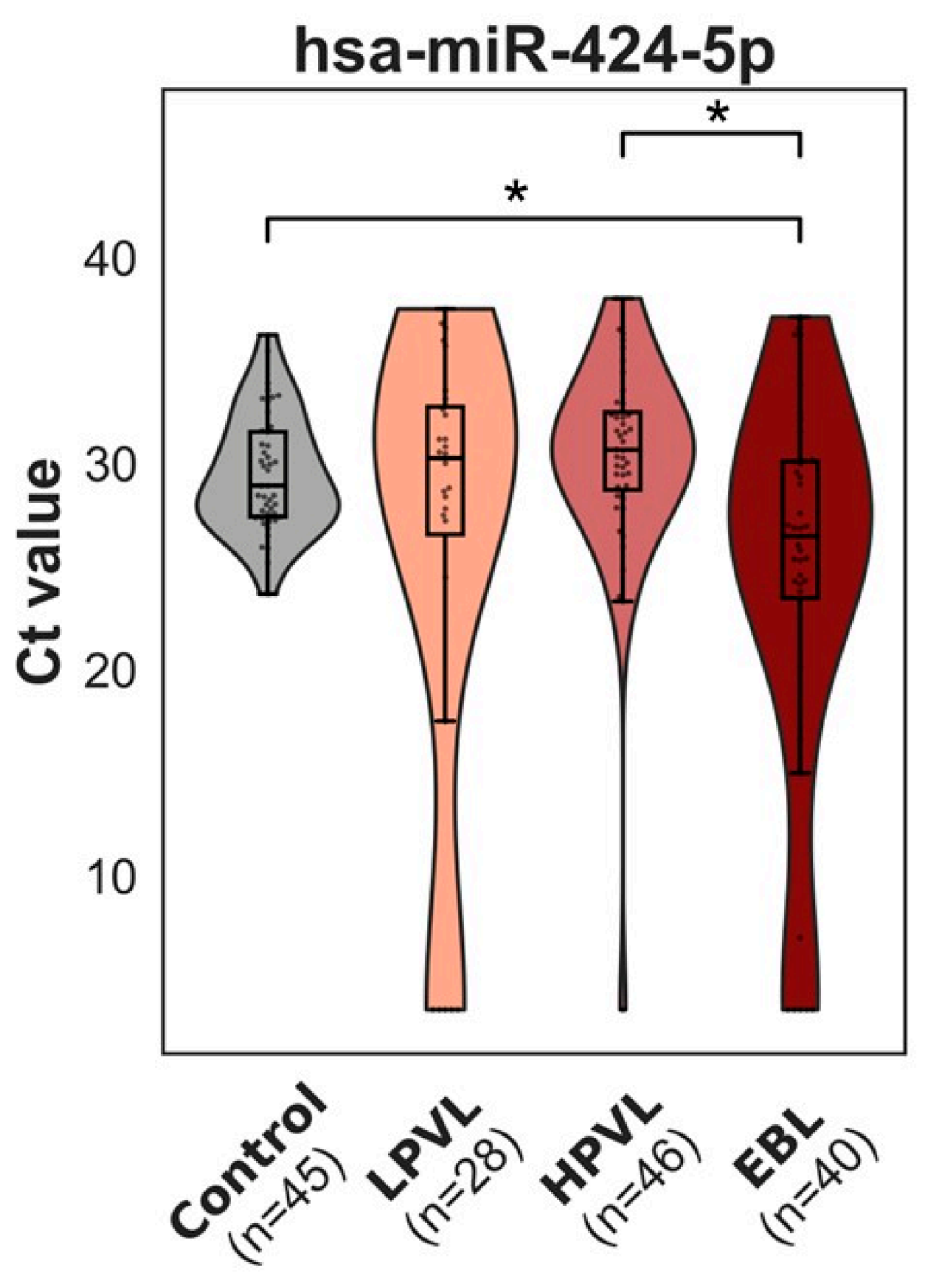
qPCR analysis of hsa-miR-424-5p in milk small extracellular vesicles (sEVs). Violin plots showing the distribution of qPCR cycle threshold (Ct) values for hsa-miR-424-5p in milk sEVs from the Control (*n* = 46), low-proviral-load (LPVL) (*n* = 28), high-proviral-load (HPVL) (*n* = 46), and enzootic bovine leukosis (EBL) (*n* = 40) groups. The width of each violin represents the kernel density of Ct values. Embedded boxplots indicate the median (center line) and interquartile range (IQR), with whiskers denoting data dispersion. Statistical comparisons between groups were performed as indicated by the brackets; * *p* < 0.05. Lower Ct values reflect higher relative miRNA abundance.

**Figure 4:**
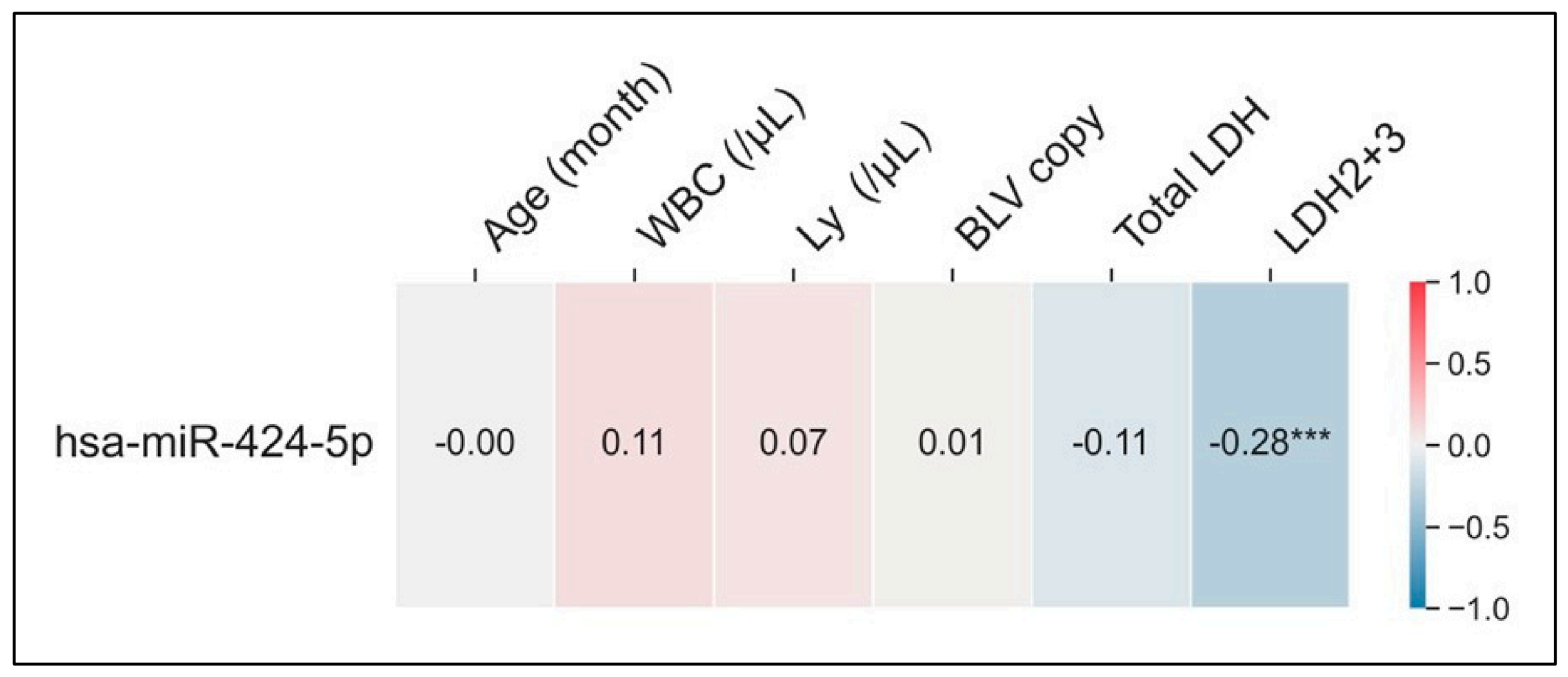
Correlation analysis of hsa-miR-424-5p in milk small extracellular vesicles (sEVs). Heatmap illustrating the correlation coefficients between hsa-miR-424-5p (Ct values) and hemato-serological variables, including age (months), white blood cell (WBC) count, lymphocyte (Ly) count, bovine leukemia virus (BLV) copies, total lactate dehydrogenase (LDH), and LDH 2 + 3%. The color intensity represents the direction and strength of the correlation, as indicated by the scale bar. A significant negative correlation was observed between hsa-miR-424-5p and LDH 2 + 3% (r = −0.29, *** *p* < 0.001), whereas correlations with other parameters were weak and not statistically significant.

**Figure 5:**
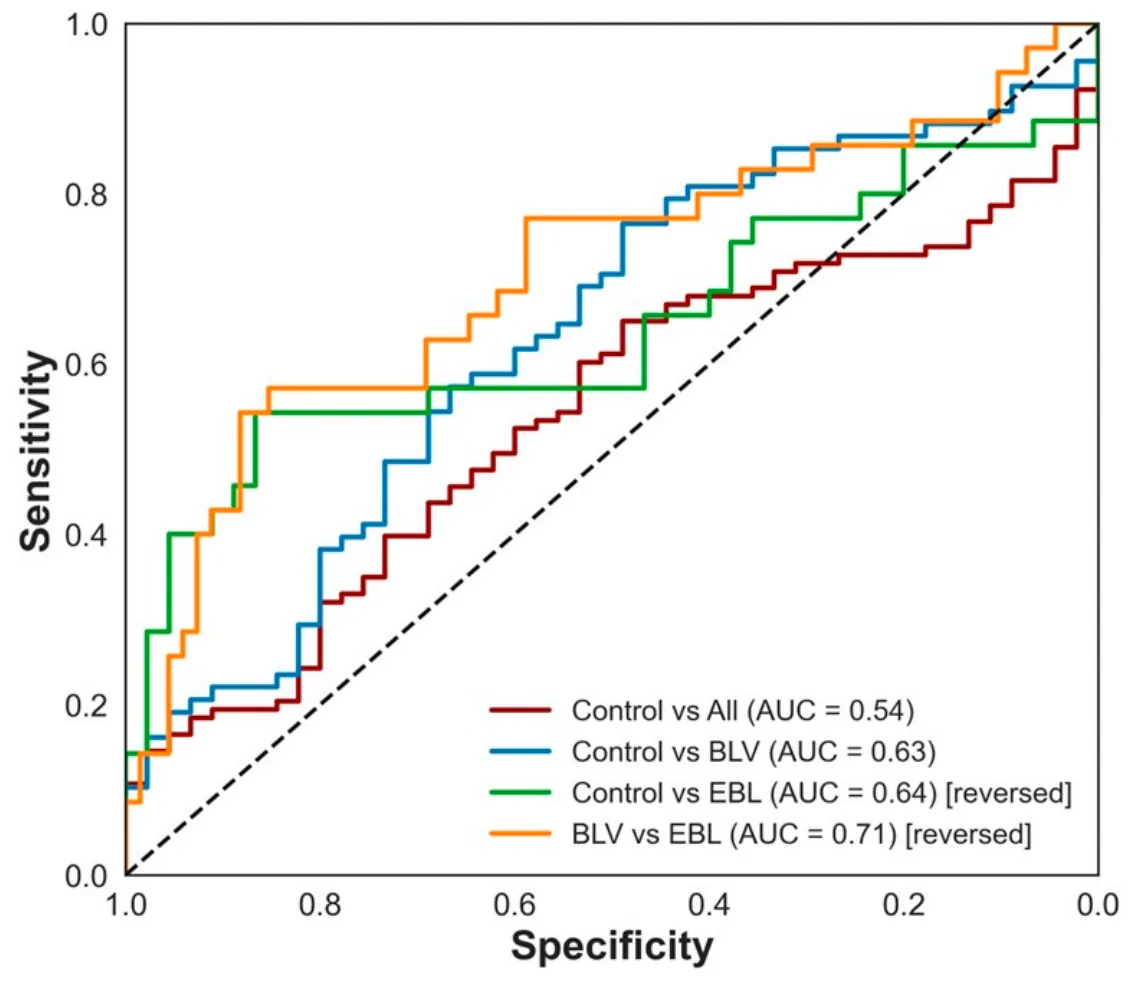
Receiver operating characteristic (ROC) curve analysis of hsa-miR-424-5p in milk small extracellular vesicles (sEVs). ROC curves evaluating the prognostic performance of hsa-miR-424-5p based on Ct values in different pairwise comparisons: Control vs. all bovine leukemia virus (BLV)-affected animals (area under the curve [AUC] = 0.54), Control vs. BLV-infected animals (AUC = 0.63), Control vs. enzootic bovine leukosis (EBL) cases (AUC = 0.64), and BLV-infected vs. EBL animals (AUC = 0.71). The dashed diagonal line represents the reference line of no discrimination (AUC = 0.50). The AUC was used as the primary measure of discriminative accuracy, with values approaching 1.0 indicating superior classification performance.

## Data Availability

All data generated or analyzed during this study are included in the manuscript and Supplementary Files.

## Supplementary Materials

The following supporting information can be downloaded at https://www.mdpi.com/article/10.3390/ani16192976/s1, Table S1: Clinical status of cattle used in this study; Figure S1: Full blot images of sEV surface marker protein CD63 and sEVs internal marker protein HSP70 (both marker proteins were successfully detected in milk sEVs from Control cattle (C1–C3; left panel) and BLV-infected cattle (B1–B3; right panel)); Figure S2: Pearson correlation between the cycle threshold values of has-miR-424-5p in milk sEVs and other variables (correlations were evaluated between hsa-miR-424-5p Ct values and (A) age, (B) total white blood cell (WBC) count, (C) lymphocyte (Ly) count, (D) BLV proviral copy number, (E) total lactate dehydrogenase (LDH) activity, and (F) LDH 2 + LDH 3% isozymes; the Pearson correlation coefficient (r) and *p*-value are shown in each panel).
