# Supplementary material for: Clinical Stage-Specific Alteration of hsa-miR-424-5p in Milk Small Extracellular Vesicles from Bovine Leukemia Virus Infection to Progression of Enzootic Bovine Leukosis
Source: Animals (Basel). 2026 Sep 22;16(19):2976. doi: 10.3390/ani16192976 (PMC13663456; doi:10.3390/ani16192976)
Supplement: Supplementary file 1 [file animals-16-02976-s001.zip › Figure S1.pdf]

Surface-marker CD63

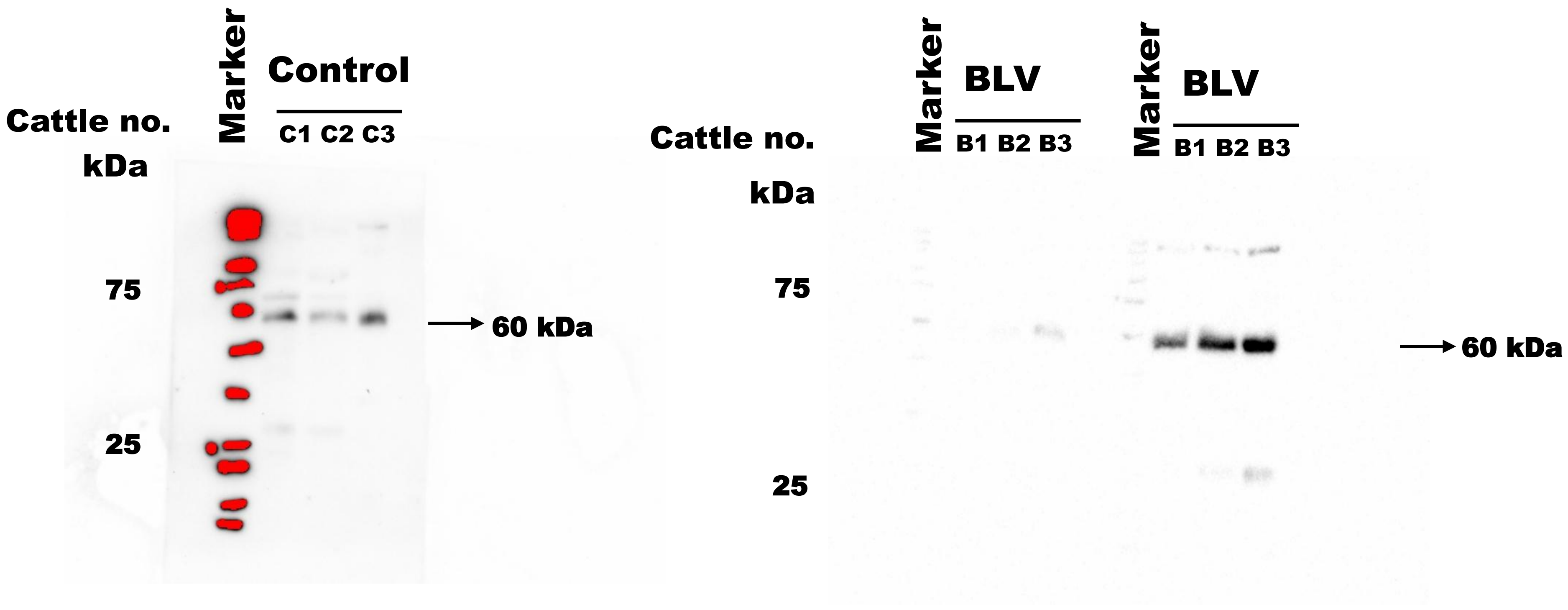

Figure S1. Full blot images of sEV surface marker protein CD63 and sEVs internal marker protein HSP70 (both marker proteins were successfully detected in milk sEVs from Control cattle (C1-C3; left panel) and BLV-infected cattle (B1-B3; right panel))

Internal-marker HSP70

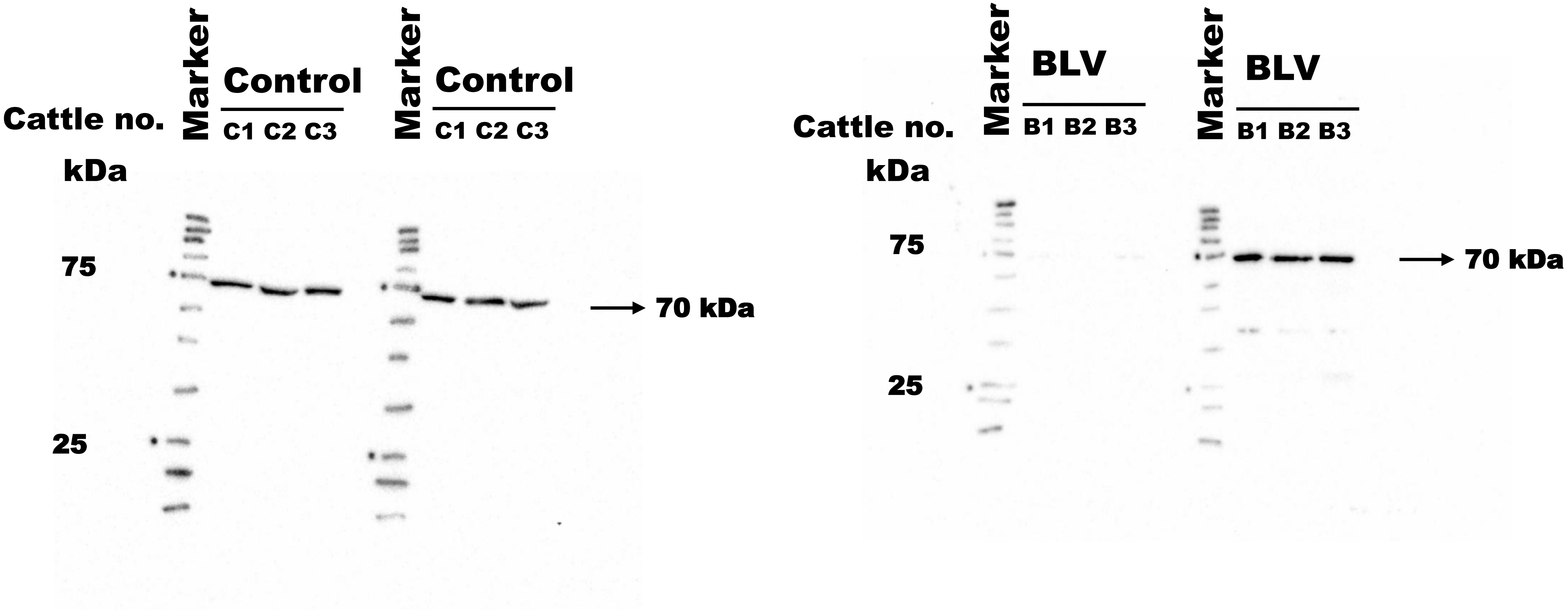

Figure S1
