# Supplementary material for: Clinical Stage-Specific Alteration of hsa-miR-424-5p in Milk Small Extracellular Vesicles from Bovine Leukemia Virus Infection to Progression of Enzootic Bovine Leukosis
Source: Animals (Basel). 2026 Sep 22;16(19):2976. doi: 10.3390/ani16192976 (PMC13663456; doi:10.3390/ani16192976)
Supplement: Supplementary file 1 [file animals-16-02976-s001.zip › Figure S2.pdf]

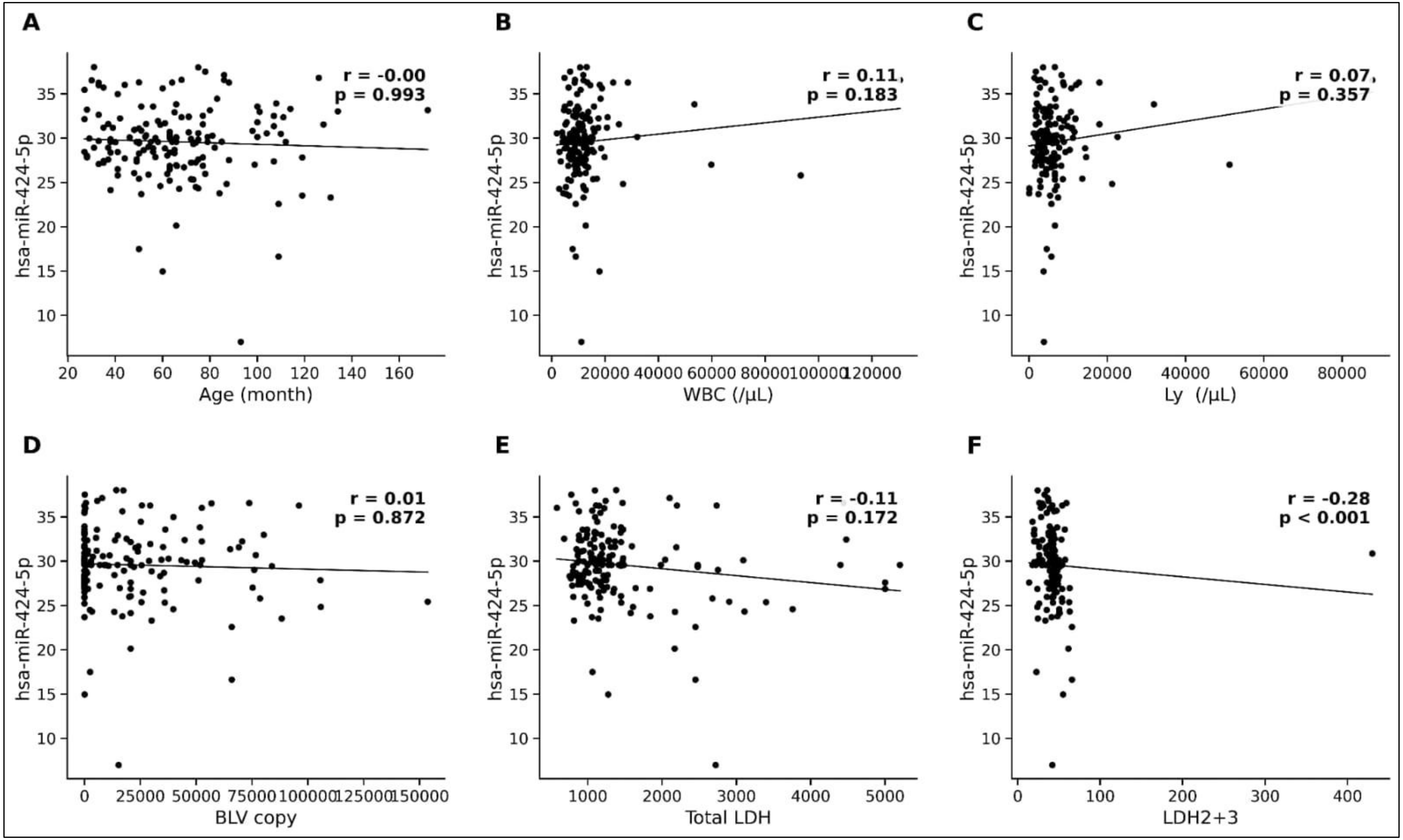

Figure S2. Pearson correlation between the cycle threshold values of has-miR-424-5p in milk sEVs and other variables
