# Supplementary material for: Clinical Stage-Specific Alteration of hsa-miR-424-5p in Milk Small Extracellular Vesicles from Bovine Leukemia Virus Infection to Progression of Enzootic Bovine Leukosis
Source: Animals (Basel). 2026 Sep 22;16(19):2976. doi: 10.3390/ani16192976 (PMC13663456; doi:10.3390/ani16192976)
Supplement: Supplementary file 1 [file animals-16-02976-s001.zip › Table S1. Clinical status of cattle.pdf]

Table S1. Clinical status of cattle used in this study.

SL. No.	Age (month)	WBC (/μL)	LY (/μL)	LY (%)	Nested-PCR	BLV copies/10^5 WBC	Total LDH (U/L)	LDH (%)					
								1	2	3	2+3	4	5
Control cattle													
1	28	9500	4600	48.3	−	−	1443	56.5	23.2	14.0	37.2	3.9	2.3
2	33	8600	4100	47.8	−	−	1153	58.5	18.7	12.4	31.1	6.0	4.4
3	27	10800	5700	53.2	−	−	1169	53.3	21.3	13.9	35.2	6.4	5.1
4	29	12200	6300	51.8	−	−	946	60.6	15.7	14.3	30.0	5.8	3.6
5	37	10200	4900	47.9	−	−	1366	63.6	14.4	11.7	26.1	5.7	4.6
6	49	7100	4000	49.4	−	−	1159	56.4	21.7	13.9	35.6	5.2	2.8
7	63	7200	2800	39.5	−	−	996	65.0	15.1	10.7	25.8	5.0	4.2
8	66	6200	1500	63.8	−	−	1355	65.7	11.9	11.8	23.7	6.1	4.5
9	74	12100	3100	25.4	−	−	1185	53.8	27.3	12.5	39.8	4.2	2.2
10	100	4200	1600	65.3	−	−	1206	67.5	14.9	10.6	25.5	4.3	2.7
11	128	5100	1700	32.6	−	−	1202	56.4	23.8	10.6	34.4	5.3	3.9
12	134	6300	3600	57.5	−	−	991	49.6	28.8	14.5	43.3	4.3	2.8
13	114	10100	3900	32.6	−	−	1095	54.4	27.6	13.5	41.1	3.3	1.2
14	100	4900	2500	50.8	−	−	992	29.0	32.3	24.7	57.0	7.7	6.3
15	108	6800	2000	29.7	−	−	1350	52.8	25.5	13.3	38.8	5.0	3.4
16	100	5600	2500	44.8	−	−	1190	54.5	23.8	13.9	37.7	5.4	2.4
17	104	1900	1200	64.0	−	−	1292	51.4	23.8	14.2	38.0	6.5	4.1
18	107	8100	1800	21.8	−	−	1087	47.2	27.2	16.7	43.9	5.9	3.0
19	172	4600	1600	34.9	−	−	1093	53.5	26.2	13.9	40.1	4.5	1.9
20	27	9800	4400	44.5	−	−	1269	47.0	27.0	16.0	43.0	6.0	4.0
21	50	13100	3500	26.9	−	−	935	41.0	27.0	23.0	50.0	7.0	2.0
22	54	8400	4400	51.9	−	−	1164	48.0	27.0	15.0	42.0	6.0	4.0
23	37	9900	4300	42.9	−	−	988	50.0	25.0	15.0	40.0	6.0	4.0
24	35	10300	5800	56.1	−	−	1026	49.0	26.0	15.0	41.0	6.0	4.0
25	107	3400	1600	47.2	−	−	911	45.0	26.0	15.0	41.0	8.0	6.0
26	38	9900	3700	37.5	−	−	1064	44.0	29.0	17.0	46.0	6.0	4.0
27	28	7500	2700	36.4	−	−	927	45.0	28.0	17.0	45.0	6.0	4.0
28	33	12000	5800	48.3	−	−	1261	51.0	27.0	13.0	40.0	5.0	4.0
29	49	10200	5000	48.9	−	−	867	38.0	29.0	20.0	49.0	8.0	5.0
30	56	2900	1100	39.2	−	−	944	44.0	28.0	16.0	44.0	7.0	5.0
31	35	10800	5700	52.4	−	−	1026	45.0	27.0	17.0	44.0	7.0	4.0
32	51	5400	2400	44.4	−	−	1057	49.0	26.0	15.0	41.0	6.0	4.0
33	41	9600	4500	46.6	−	−	1147	42.0	29.0	18.0	47.0	7.0	4.0
34	28	11700	5700	48.5	−	−	1106	50.0	28.0	14.0	42.0	5.0	3.0
35	31	8600	3900	45.1	−	−	1108	47.0	25.0	16.0	41.0	7.0	5.0
36	44	8300	2800	34.0	−	−	1183	38.0	25.0	17.0	42.0	9.0	11.0
37	62	6500	2900	44.1	−	−	774	46.0	27.0	16.0	43.0	7.0	4.0
38	37	12000	3900	32.2	−	−	1149	43.0	30.0	18.0	48.0	6.0	3.0
39	62	10500	3400	32.3	−	−	960	48.0	28.0	15.0	43.0	5.0	4.0
40	63	8600	1400	16.1	−	−	1213	43.0	29.0	18.0	47.0	6.0	4.0
41	51	10000	4200	42.4	−	−	1047	45.0	28.0	15.0	43.0	6.0	6.0
42	48	8300	4500	54.6	−	−	1032	45.0	25.0	16.0	41.0	8.0	6.0
43	53	8700	3600	41.7	−	−	1081	45.0	29.0	16.0	45.0	6.0	4.0
44	46	8400	4700	56.4	−	−	1152	50.0	28.0	14.0	42.0	5.0	3.0
45	33	7400	3800	51.1	−	−	1141	47.0	27.0	15.0	42.0	6.0	5.0

+, positive; −, negative; NT, not tested; BLV, bovine leukemia virus; LY, lymphocytes; nested-PCR, nested-polymerase chain reaction; WBC, white blood cell; LDH, lactate dehydrogenase

Table S1. (continued)

SL. No.	Age (month)	WBC (/μL)	LY (/μL)	LY (%)	Nested-PCR	BLV copies/10^5 WBC	Total LDH (U/L)	LDH (%)					
								1	2	3	2+3	4	5
LPVL cattle (< 10,000 DNA copies/105 WBCs)													
1	112	5300	2400	44.8	+	4	1116	44.2	31.8	17.2	49.0	4.6	2.2
2	78	6900	1700	NT	+	79	778	62.5	19.8	12.6	32.4	3.5	1.6
3	80	8800	1800	20.5	NT	102	1117	72.0	11.0	8.7	19.7	4.5	3.8
4	33	14400	8900	61.9	NT	332	1222	56.6	24.8	14.1	38.9	3.6	0.0
5	68	8700	2900	NT	+	596	1469	69.2	15.8	10.5	26.3	3.0	1.5
6	50	7200	3300	45.3	+	1037	1418	69.6	10.2	11.7	21.9	5.1	3.4
7	119	6500	2200	33.1	+	1063	1109	46.7	20.4	21.5	41.9	5.8	5.6
8	111	8900	4000	45.3	+	1186	1284	47.9	25.2	15.1	40.3	6.8	5.0
9	98	11900	3100	26.3	+	1367	1188	56.3	22.6	12.7	35.3	5.4	3.0
10	74	5300	2900	55.3	+	2402	1175	56.7	21.4	13.7	35.1	4.9	3.3
11	50	7800	4500	57.8	+	2476	1062	69.5	9.4	13.3	22.7	4.8	3.0
12	38	7900	3200	40.1	+	3062	1193	71.4	7.4	10.4	17.8	5.7	5.1
13	126	4800	1400	29.6	+	5732	1239	56.9	24.8	12.4	37.2	4.7	1.2
14	49	9300	5300	57.0	+	6821	1318	51.3	23.1	13.5	36.6	7.0	5.1
15	50	7800	4500	57.8	+	8751	1111	57.2	20.8	13.3	34.1	5.8	2.9
16	75	7400	4800	64.8	+	8184	1166	40.0	24.0	15.0	39.0	9.0	12.0
17	110	6800	2800	40.5	+	3742	1047	39.0	27.0	19.0	46.0	8.0	7.0
18	35	20700	8300	40.2	+	1525	944	45.0	27.0	15.0	42.0	6.0	7.0
19	35	6300	2800	45.0	+	23	1060	42.0	29.0	18.0	47.0	6.0	5.0
20	51	9600	5400	56.2	+	5538	931	47.0	27.0	16.0	43.0	6.0	4.0
21	39	17400	11000	63.4	+	5448	864	45.0	29.0	16.0	45.0	6.0	4.0
22	38	13400	3100	23.0	+	256	839	47.0	28.0	15.0	43.0	6.0	4.0
23	52	6800	3100	45.5	+	1163	842	48.0	27.0	15.0	42.0	6.0	4.0
24	33	11500	4300	37.3	+	5133	983	47.0	27.0	15.0	42.0	6.0	5.0
25	88	9100	4000	44.1	+	164	919	49.0	28.0	14.0	42.0	5.0	4.0
26	72	11600	5500	47.8	+	6721	793	49.0	26.0	14.0	40.0	6.0	5.0
27	61	4900	2600	53.1	+	842	802	47.0	28.0	15.0	43.0	6.0	4.0
28	57	6500	1900	29.9	+	Below detection limit	1048	47.0	28.0	15.0	43.0	5.0	5.0

+, positive; −, negative; NT, not tested; BLV, bovine leukemia virus; LY, lymphocytes; nested-PCR, nested-polymerase chain reaction; WBC, white blood cell; LDH, lactate dehydrogenase

Table S1. (continued)

SL. No.	Age (month)	WBC (/μL)	LY (/μL)	LY (%)	Nested-PCR	BLV copies/10^5 WBC	Total LDH (U/L)	LDH (%)					
								1	2	3	2+3	4	5
HPVL cattle (≥ 10,000 DNA copies/105 WBCs)													
1	31	13100	6600	50.1	+	14238	1383	56.0	21.5	14.0	35.5	4.9	3.6
2	75	10600	3600	34.4	+	17357	1097	65.9	13.3	10.9	24.2	5.2	4.7
3	61	10100	5000	50.0	+	24114	1595	66.6	17.0	9.0	26.0	4.4	3.0
4	83	18300	6700	36.6	+	25154	1128	77.2	8.8	9.2	18.0	2.9	1.9
5	56	15800	6800	42.9	+	36185	1477	75.8	12.8	7.4	20.2	2.3	1.7
6	72	10300	6500	63.6	+	45748	1141	71.0	9.5	10.7	20.2	5.3	3.5
7	49	14800	8900	60.4	+	51781	1280	54.2	23.2	11.9	35.1	3.6	7.1
8	41	9500	1900	28.3	NT	39799	1098	64.8	17.0	11.3	28.3	3.4	3.5
9	65	9200	5500	37.8	NT	14830	883	55.0	23.9	13.9	37.8	4.2	3.0
10	40	7700	4800	29.4	+	25524	996	60.5	16.8	12.6	29.4	6.0	41.0
11	78	8800	4200	35.4	+	20045	807	57.6	23.2	12.2	35.4	4.4	26.0
12	131	11900	7400	33.4	+	29977	815	53.8	18.0	15.4	33.4	7.9	49.0
13	71	13300	5400	29.6	+	44845	883	62.9	18.0	11.6	29.6	4.6	2.9
14	44	18000	12000	27.9	NT	52469	584	70.4	18.2	9.7	27.9	1.4	30.0
15	42	12500	7700	20.0	+	52505	1274	67.1	9.1	10.9	20.0	6.2	6.7
16	30	11900	2200	29.4	NT	56843	838	65.4	18.7	10.7	29.4	3.1	2.1
17	107	11600	9800	33.8	NT	65196	848	61.1	22.5	11.3	33.8	3.1	2.0
18	42	17500	11800	23.0	+	70620	1366	66.3	12.9	10.3	23.2	5.4	5.1
19	63	16000	11600	26.0	+	76662	1331	67.1	15.0	11.0	26.0	4.3	2.6
20	101	13700	10700	78.2	+	80205	1185	69.7	8.7	11.3	20.0	5.2	5.1
21	119	6500	5400	83.4	+	88285	1143	68.0	15.1	9.3	24.4	4.2	3.4
22	87	26700	21200	79.4	+	105784	1608	67.8	11.9	11.8	23.7	5.2	3.3
23	50	15600	10900	69.6	+	23851	916	46.0	28.0	16.0	44.0	6.0	4.0
24	59	10000	5500	55.4	+	22732	1091	42.0	26.0	17.0	43.0	8.0	7.0
25	63	12300	7000	56.7	+	18764	1477	32.0	23.0	18.0	41.0	11.0	16.0
26	82	13100	7700	59.1	+	17617	973	48.0	25.0	14.0	39.0	7.0	6.0
27	60	18800	10800	57.3	+	16924	882	44.0	28.0	16.0	44.0	7.0	5.0
28	68	20900	9800	47.0	+	13681	916	49.0	27.0	14.0	41.0	6.0	4.0
29	41	15700	9300	59.5	+	13404	892	41.0	27.0	18.0	45.0	7.0	7.0
30	71	3900	2600	65.4	+	11251	863	41.0	25.0	18.0	43.0	9.0	7.0
31	58	14300	8900	62.6	+	29482	1031	45.0	28.0	15.0	43.0	6.0	6.0
32	50	13300	8700	65.1	+	25696	1112	47.0	27.0	15.0	42.0	6.0	5.0
33	56	18500	14300	77.1	+	30004	968	47.0	29.0	15.0	44.0	5.0	4.0
34	77	9200	4800	52.5	+	18779	683	44.0	29.0	16.0	45.0	6.0	5.0
35	63	15300	11200	73.5	+	28911	882	47.0	28.0	15.0	43.0	6.0	5.0
36	65	10300	6400	62.4	+	33874	757	50.0	27.0	14.0	41.0	5.0	4.0
37	81	9500	6300	66.6	+	37624	1058	47.0	28.0	15.0	43.0	6.0	4.0
38	75	9900	5300	53.5	+	49424	860	46.0	28.0	16.0	44.0	6.0	4.0
39	77	12000	7400	61.5	+	51017	883	44.0	29.0	17.0	46.0	6.0	4.0
40	57	19800	14600	73.9	+	105564	1247	40.0	30.0	19.0	49.0	7.0	4.0
41	65	15100	10400	68.7	+	35553	1011	48.0	27.0	15.0	42.0	6.0	4.0
42	74	NT	NT	NT	+	10754	NT	NT	NT	NT	NT	NT	NT
43	53	11400	6700	58.4	NT	35175	1311	50.0	25.0	14.0	39.0	6.0	5.0
44	50	10400	6000	57.4	NT	83888	805	47.0	28.0	15.0	43.0	6.0	4.0
45	69	13600	5700	41.7	NT	35693	1214	47.0	27.0	17.0	44.0	5.0	4.0
46	63	9000	4400	49.3	NT	26249	1345	46.0	30.0	15.0	45.0	5.0	4.0

+, positive; −, negative; NT, not tested; BLV, bovine leukemia virus; LY, lymphocytes; nested-PCR, nested-polymerase chain reaction; WBC, white blood cell; LDH, lactate dehydrogenase

Table S1. (continued)

SL. No.	Age (month)	WBC (/μL)	LY (/μL)	LY (%)	Nested-PCR	BLV copies/10^5 WBC	Total LDH (U/L)	LDH (%)					
								1	2	3	2+3	4	5
EBL cattle													
1	53	8400	4956	73.0	+	34383	2042	35.8	36.6	20.9	57.5	5.3	1.4
2	38	NT	NT	57.0	NT	NT	1577	42.8	33.2	17.7	50.9	5.0	1.3
3	73	8800	2649	30.1	+	22146	2479	29.5	29.2	21.5	50.7	9.2	10.6
4	73	6100	NT	53.7	+	35949	1450	42.7	25.5	17.2	42.7	6.8	7.8
5	NT	NT	NT	NT	NT	NT	2169	29.8	37.8	23.6	61.4	7.0	1.8
6	99	59800	51249	85.7	+	75270	1046	48.0	25.8	18.3	44.1	5.7	2.2
7	55	NT	NT	NT	NT	NT	1488	44.0	27.2	19.7	46.9	6.6	2.5
8	79	NT	NT	NT	NT	NT	4400	41.3	36.5	17.2	53.7	3.8	1.2
9	75	NT	NT	NT	NT	NT	5000	7.9	7.5	6.3	13.8	7.0	71.3
10	27	14200	11789	NT	NT	NT	NT	NT	NT	NT	NT	NT	NT
11	48	NT	NT	NT	NT	NT	NT	NT	NT	NT	NT	NT	NT
12	ND	over	ND	ND	+	2898	1471	38.9	19.5	19.8	39.3	12.4	9.4
13	ND	8500	3200	38.2	NT	15792	1645	22.5	3.8	59.1	62.9	8.9	5.7
14	41	93300	ND	ND	+	78549	2678	37.4	34.9	18.7	53.6	6.5	2.5
15	77	25200	18000	71.6	+	69048	2193	60.3	24.4	10.0	34.4	3.4	1.9
16	67	2800	100	5.1	+	13306	2173	35.5	29.4	21.1	50.5	5.8	8.2
17	109	9000	5700	63.6	+	65943	2448	25.1	40.7	25.1	65.8	7.0	2.1
18	86	130500	87700	67.2	+	73756	4442	23.0	32.5	26.4	58.9	6.9	11.2
19	72	15500	8600	55.8	+	25435	3399	28.3	36.2	26.2	62.4	8.0	1.3
20	77	9500	8200	86.6	NT	ND	1838	49.5	25.4	12.6	38.0	5.5	7.0
21	63	Over	NT	NT	+	1580	5000	41.3	38.2	15.7	53.9	3.8	1.0
22	85	6900	2600	38.2	+	14774	1321	45.3	26.1	17.0	43.1	6.9	4.7
23	54	5500	2200	39.6	+	75934	2750	51.3	31.0	12.3	43.3	2.8	2.6
24	86	11800	8300	70.5	+	7893	2102	43.4	24.9	11.6	36.5	2.9	17.2
25	84	4300	11	26.0	+	16947	1842	44.3	29.3	18.9	48.2	5.7	1.8
26	75	12000	26	21.9	+	3321	3109	28.0	35.9	27.0	62.9	5.5	3.6
27	50	16900	13600	80.4	+	153632	2903	40.2	36.2	17.6	53.8	4.1	1.9
28	59	11800	7100	60.5	+	39692	3757	45.4	36.2	14.0	50.2	3.4	1.0
29	ND	11300	7100	62.8	+	NT	5201	13.0	10.0	8.0	18.0	11.0	58.0
30	88	28500	18000	63.0	+	29378	2734	27.4	28.8	13.4	42.2	6.0	24.4
31	64	23000	12700	55.2	+	96045	2201	36.0	31.8	22.0	53.8	7.6	2.6
32	ND	14800	8400	56.7	+	43327	3092	27.7	15.9	13.7	29.6	8.3	34.4
33	ND	53500	31900	59.6	+	51671	>5000	52.0	43.4	4.4	47.8	0.2	0.0
34	58	4000	2700	67.8	+	9566	2479	41.0	34.8	19.0	53.8	4.1	1.1
35	109	9000	5700	63.6	+	65943	2448	25.1	40.7	25.1	65.8	7.0	2.1
36	79	32000	22600	70.7	+	52587	1283	31.0	28.0	22.0	50.0	11.0	8.0
37	71	11500	1900	16.2	+	0	4479	32.0	33.0	20.0	53.0	9.0	6.0
38	64	13600	5800	42.9	+	535	1985	26.0	29.0	25.0	54.0	14.0	6.0
39	93	11100	3800	34.2	+	15236	2721	20.0	23.0	19.0	42.0	9.0	29.0
40	60	17900	3700	20.4	+	69	1274	34.0	34.0	21.0	55.0	7.0	4.0

+, positive; −, negative; NT, not tested; BLV, bovine leukemia virus; LY, lymphocytes; nested-PCR, nested-polymerase chain reaction; WBC, white blood cell; LDH, lactate dehydrogenase
